# Supplementary material for: Productivity losses and treatment cost of benign prostatic hyperplasia in Ghana
Source: PLoS One. 2026 Jan 22;21(1):e0341280. doi: 10.1371/journal.pone.0341280 (PMC12826523; doi:10.1371/journal.pone.0341280)
Supplement: Attachment — Submitted filename: response to reviewers.docx [file pone.0341280.s002.docx]

Department of Surgery

School of Medicine

University of Health and Allied Sciences

29^th^ December 2025

The Editor

PLOS One Journal

Dear Editor,

**RESPONSE TO REVIEWERS COMMENTS**

On behalf of my co-authors, I write in response to comments made by reviewers on our study titled “Productivity losses and treatment cost of Benign Prostatic Hyperplasia in Ghana.” We deeply appreciate the reviewer comments, which have significantly enriched the quality of our work.

Please see below the comments and their responses.

| Comments | Responses |
| --- | --- |
| Reviewer 1 |  |
| 1. The study addresses an important evidence gap—the economic burden of BPH in Ghana. However, data from a single tertiary hospital may not represent the broader population. Patients at referral centers often have more advanced disease or greater financial means to access specialist care. The discussion could better acknowledge how these selection factors may bias cost estimates and limit national generalizability. | The comment is well noted and accepted. The discussion section has been revised to reflect this important limitation. Please see page 19. |
| 2. The discussion references some studies from Europe and Asia but does not include countries of similar economic status to Ghana. Even brief inclusion of such studies would contextualize Ghana's results within a comparable economic and healthcare framework. | This suggestion has been accepted and the manuscript edited accordingly. Studies from Nigeria, Kenya and Uganda have been cited. |
| 3. The manuscript’s main message is that BPH imposes a “significant economic burden,” but it lacks a clear linkage to policy or health system strategies (e.g., screening, early intervention, insurance coverage). Expanding this section to describe how the results could inform local reimbursement or urological service planning would strengthen its relevance. | Well accepted. We have added a paragraph on how the results could influence policy. Please see page 19. |
| Reviewer 2 |  |
| Major comments |  |
| 1. BPH disease severity was not measured or adjusted, despite being a key driver of treatment choice and cost. This is a major limitation that should be emphasized. | Well acknowledged. We have edited the limitation section to include the lack of assessment of disease severity as a major limitation. Page 19 & 20. |
| 2. Productivity loss estimation using minimum wage for all participants likely underestimates indirect costs and ignores income heterogeneity. | This criticism is well accepted. We have acknowledged this as a notable limitation in the manuscript. Page 19 |
| 3. Catastrophic health expenditure (CHE) appears to be calculated using mean costs, rather than individual patient-level cost–income ratios, which may misclassify CHE. If feasible, re-estimate CHE using individual patient-level cost–income ratios. | We welcome the suggestion, however it may not be feasible since income levels in this study were determined using income brackets (eg < GHS 500, GHS 500 – GHS 1,999 etc) rather than ascertaining individual incomes. Due to this constraint, the mid-point income level was used. |
| 4. Regression analysis is under-specified, excluding key clinical and treatment-related variables. Regression findings should be reframed as exploratory, and the limitations imposed by omitted variables should be explicitly discussed. | The regression analysis included variables such as income levels, educational and marital status. We admit that key clinical variables such as disease severity were omitted. The discussion section has been edited accordingly. Page 18. |
| Minor comments |  |
| 1. Costs are reported in both GHS and USD using a single exchange rate. The authors should clarify whether any inflation adjustment was applied and state explicitly that USD conversions are for international comparison | No inflation adjustments were made. We have included that the USD conversions were meant for international comparisons. Page 10. |
| 2. Standardize terminology for indirect costs and productivity losses throughout the manuscript. | We have accepted the recommendation and the manuscript has been edited accordingly. |
| 3. Large standard deviations suggest skewed cost data; A brief justification for reporting means alongside medians would improve statistical clarity. | A justification has been provided. Please see page 10. |
| 4. Strengthen policy implications: More concrete policy recommendations (e.g., NHIS medication coverage expansion, decentralization of urology services, transport subsidies) would strengthen the public health relevance. | A paragraph on policy implications has been added. Please see page 19. |

Yours sincerely,

………………………..

Dr. Kekeli Kodjo Adanu

Corresponding author
